# Cardiopulmonary response during incremental shuttle walking test in a hallway versus on treadmill in Phase IV cardiac rehabilitation: a cross-sectional study

**DOI:** 10.1038/s41598-023-39999-2

**Published:** 2023-08-07

**Authors:** Ahmad M. Osailan

**Affiliations:** Department of Physical Therapy and Health Rehabilitation, College of Applied Medical Sciences, Prince Sattam Bin Abdulaziz University, Alkharj, Saudi Arabia

**Keywords:** Physiology, Cardiology, Health care, Risk factors

## Abstract

There is widespread use of incremental shuttle walking test (ISWT) to measure functional capacity in cardiac rehabilitation patients. Due to occasional physical space limitations, an incremental shuttle walking test on a treadmill (ISWT-T) was suggested as an alternative. Knowledge about the cardiopulmonary response between the two tests and the factors associated with the distance achieved in Phase IV cardiac rehabilitation is limited. Thus, the study aims to compare the cardiopulmonary response between ISWT and ISWT-T and investigate the factors associated with distance achieved in both tests. Thirteen participants (66.3 ± 7.3 years, 84.6% males) attending phase IV cardiac rehabilitation participated in repeated measures counterbalanced trials. Each participant performed one ISWT and one ISWT-T separated by seven days. Main outcome measures included peak heart rate (HR), systolic and diastolic blood pressure post-test, distance achieved, respiratory frequency, tidal volume (VT), minute ventilation, respiratory exchange ratio, peak oxygen uptake (VO_2PEAK_), and secondary outcome measures included height, weight, waist circumference (WC) leg length (LL). There were no significant differences in the cardiopulmonary responses between ISWT and ISWT-T except for VO_2PEAK_ (25.4 ± 5.8 vs 23.7 ± 5.1, p = 0.05, respectively). Age and height were significantly correlated with distance achieved during ISWT, and ISWT-T [age (*r* = − 0.72, vs. *r* = − 0.73, *p* ≤ 0.05, respectively)], [height (*r* = 0.68, vs. *r* = 0.68, *p* ≤ 0.05, respectively)]. LL was only correlated with distance achieved on ISWT-T (*r* = 0.59, *p* ≤ 0.05). These findings suggest a similar cardiopulmonary response between the two tests, but doing ISWT in the hallway evoked a higher metabolic demand than doing it on a treadmill. Additionally, distance achieved on both tests was related to height and inversely to age.

## Introduction

The incremental shuttle walking test (ISWT) was developed as a field test to measure functional capacity in patients with chronic obstructive pulmonary diseases^1^. ISWT is incremental and progressive to provoke symptoms limited maximal performance. Due to its reliability and sensitivity to changes in functional capacity^1,2^, it has been widely used in different clinical populations, including people with cardiovascular diseases (CVD). The test is simple to administer and requires a 10 m course on a flat surface marked with two cones, while audible bleeps dictate the pace with an increment at one minute. However, finding an appropriate physical space to conduct the test is sometimes challenging. To overcome this issue, ISWT was suggested to be performed on a treadmill (ISWT-T).

An assumption was that performing ISWT-T would promote similar walking distances and physiological responses similar to ISWT performed in the hallway^3^. Indeed, a study found no differences in walking distance and other physiological responses between ISWT and ISWT-T^4^. On the contrary, It was argued that when field tests were performed in a hallway and on a treadmill, there was a reduction in walking distances achieved on the treadmill, including 6-min walk test (6MWT)^5^, and 12-min walk test and ISWT^6^. Also, it was found that there was a higher energy cost per meter with ISWT-T among cardiac rehabilitation patients at the early stages of the test^7^. Regarding gait mechanics between walking on a treadmill and in a hallway, there might be some critical differences that may result in variations in physiological responses. For example, greater energy is needed to maintain balance during walking on a treadmill, especially for those unfamiliar with it^8^.

ISWT and ISWT-T were routinely used as simple field test for assessing patients with CVD before enrollment into cardiac rehabilitation. The latter was used as an alternative in case of limitation of physical space. However, knowledge about the physiological responses during ISWT and ISWT-T and whether it is different or similar in people attending Phase IV cardiac rehabilitation is limited. A previous study reported variation in energy demands of each level of ISWT when performed in a hallway and on a treadmill. It concluded that metabolic demands differ significantly between the two tests favouring the treadmill at lower test speeds and walking in the hallway at higher speeds^7^. The previous study aimed to compare the metabolic demands between the two methods (ISWT vs ISWT-T) of the tests with the estimated equations published from performing ISWT on the treadmill on healthy participants^2^. Information about the differences in cardiopulmonary responses was not reported, nor were the factors associated with the achieved distance of each test. Nevertheless, it must be noted that ISWT also provides a good alternative when physical space is not a limiting factor for testing cardiac rehabilitation patients, especially during times when physical distancing is a more favourable option especially to minimize the spread of communicable diseases such as during COVID-19 pandemic^9^.

To the best of knowledge, no study investigated the cardiopulmonary responses between performing ISWT and ISWT-T within the same participants of Phase IV cardiac rehabilitation. Knowing cardiopulmonary responses will aid the decision of whether ISWT-T can be used as an alternative to ISWT in cases of physical space limitation. Thus, the current study aimed to compare the cardiopulmonary responses between ISWT and ISWT-T. A secondary aim was to investigate the factors associated with the achieved distance of each test. It was hypothesized that both tests will produce similar physiological responses in phase IV cardiac rehabilitation attendees.

## Methods

### Participants

Thirteen participants (66.3 ± 7.3 years, 84.6% males) were recruited from a single community-based center for cardiac rehabilitation, following cardiac revascularization surgery or a confirmed diagnosis of CVD. The participants were approached by word of mouth at the community center by the investigator (the author), who also was working as the instructor during exercise sessions. A brief information sheet was also distributed to the participants and were given a week to respond. Upon agreement to participate, participants gave written, informed consent. Inclusion criteria were cardiac revascularization and confirmed diagnosis of a stable CVD. Exclusion criteria were people with musculoskeletal disorders preventing them from performing ISWT, neurological conditions (e.g., parkinsonism, dementia), unstable hypertension, unstable angina, had myocardial infarction during the previous month, uncontrolled arrhythmia, uncontrolled diabetes mellites or comorbidity incompatible with exercise testing as per American College of Sports Medicine (ACSM)^10^. A Post hoc analysis using G*power software for the current sample size (n = 13) showed that it yielded a power of (1 − β error probability) = 0.82 with an effect size of (*d*) = 0.75. The study was conducted according to the guidelines of the Declaration of Helsinki and approved by the Ethical Committee (RHPT/022/021) at Prince Sattam bin Abdulaziz University.

### Study protocol

**Figure 1:**
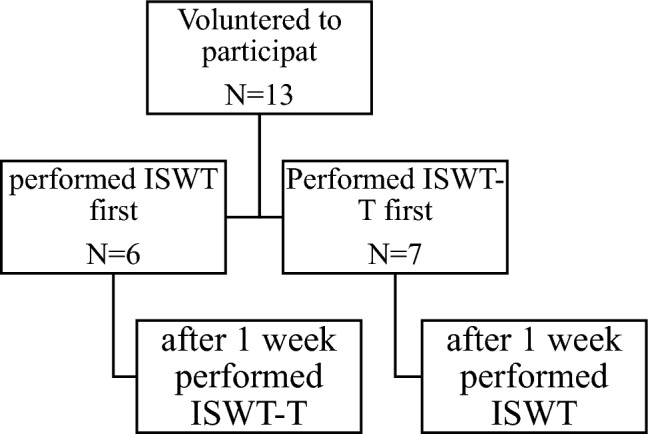
Flow chart of the study. *ISWT* Incremental shuttle walking test, *ISWT-T* incremental shuttle walking test -Treadmill.

This cross-sectional study (repeated measures with counterbalance trials) was performed in two visits (a week apart). The participants were randomized to perform ISWT or ISWT-T first. Participants visited the Biomechanics lab at the sports center at the University of Essex at 4:00 p.m. Every week two participants were called for the test. One to perform for ISWT and the other to perform ISWT-T. In the next session of the same week, the two participants alternated the test. For safety reasons, before starting the actual test, a familiarization period (two minutes at a slow speed of ~ 0.3–0.45 m/s was allowed for participants before ISWT-T to minimize miss coordination in taking steps during the actual test and loss of balance). This was done for every participant before the attachment of the face mask and polar HR monitor. There was no full practice test, as all the participants successfully walked on the treadmill and completed the speed of every progressed level. Before starting the test, height, weight, waist circumference, leg length, resting heart rate, and resting blood pressure while seated were measured using an automated blood pressure device (Wollex Blood Pressure Monitor (ARM)/WXT-5902, Cigli Izmir, Turkey). Participants were evaluated before the start of ISWT for any relative contraindication, including resting heart rate > 120 beats per minute, systolic blood pressure > 180 mmHg, and diastolic blood pressure > 100 mmHg. During the test, participants were fitted with a Polar heart rate monitor (Polar H7) and a face mask covering the nose and the mouth connected with a portable gas analyzer to measure inspired and expired gases (K4b2 Mobile Breath by Breath Metabolic System, COSMED Pulmonary Function Equipment, Rome, Italy). The portable gas analyzer was calibrated each day before the start of testing. While seated, resting metabolic rate (obtained from the volumes of O_2_ consumption), and resting heart rate were measured for three minutes, followed by either ISWT or ISWT-T. At the end of the test, a recovery period was applied for a minimum of six minutes (see Fig. 1).

#### Incremental shuttle walking test

The ISWT was performed on a non-slippery flat surface with cones set 9 m apart and according to the national recommendations for cardiac patients^11^. The total distance for the course was 10 m considering 0.5 m around each cone. Audible bleeps from the CD player controlled the speed of walking. The test was initiated after triple bleeps from the countdown, and then participants aimed to reach the opposite cone before the second single bleep. Since the first level is slow-paced, participants were accompanied by the investigator to set the correct required pace during the first level. A triple bleep marked increased speed and progression to the next level. The ISWT was composed of 12 levels lasting one minute each. The initial pace was 0.5 m/s with an increment of 0.17 m/s each minute until a maximum speed of 2.37 m/s. VO_2_, heart rate (HR), rate of perceived exertion (RPE) and any symptoms indicating volitional exhaustion were monitored during and at the end of the test. The test was terminated on completion or if any of the termination criteria were met, which included any angina symptoms or severe shortness of breath; feeling dizzy or faint; leg pain (e.g., severe cramping or "burning"); RPE ≥ 15 on the Borg scale; achieved 85% predicted HR reserve; or failure to walk at the same rate as the speed requirements of the test (i.e., more than 0.5 m from the cone when the beep sounds on two consecutive cones)^12^.

#### Incremental shuttle walking test-treadmill

The exact speed and increment protocol of the ISWT was programmed into a treadmill without inclination (Quaser, HP Cosmos, Nussdorf, Germany). Participants not familiar with walking on a treadmill were given a period of familiarization (2 min with no data recording) before the commencement of the test. Termination criteria for ISWT-T were similar to ISWT except for missing the cones criterion. If the participant struggled to keep the pace of a level and could not maintain speed, the test was terminated. The same variables measured during ISWT were also measured during ISWT-T.

### Gas exchange analysis

A portable breath-by-breath gas analyzer was used to measure the inspired and expired gases before, during the two tests, and the recovery period, including volume of oxygen (VO_2_), carbon dioxide (CO_2_), and minute ventilation (VE), and tidal volume (VT). Data for VO_2_ were averaged into each minute last 30 s of the sampling rate, and the highest VO_2_ recorded during each test was defined VO_2PEAK_ and was expressed as VO_2_ ml/kg/min.

### Anthropometric measurement

Waist circumference was measured using tape placed in the middle between the bottom of the ribs and the top of the hips just above the umbilicus while the participant was standing and breathing out. Leg length was measured using tape from the anterior superior iliac spine to the medial malleolus. Body mass index (BMI) was expressed as weight in kg divided by the square root of height (weight (kg)/[height (m)^2^).

### Distance

The distance was measured during ISWT by multiplying the number of shuttles performed by the total distance of the course (10 m). When the test was terminated, the participant was provided with a chair to sit on, and the spot was marked for distance measurement from the last cone. During ISWT-T, the distance was automatically measured and recorded from the treadmill's control display.

### Statistical analysis

Data were analyzed using Statistical Package for Social Sciences (IBM SPSS) (version 27, Armonk, NY, USA). Kolmogorov–Smirnov test was used to test the normality of the variables. All data were normally distributed and were presented as means and standard deviations. Paired *t*-test was used to compare the primary outcome measures between ISWT and ISWT-T, including VE, VT, VO_2PEAK_ and HRR1min. Pearsons's moment correlation was used to investigate the factors associated with the distance achieved during each test. The level of significance was set at *p* ≤ 0.05.

## Results

Figure 1 shows the flow chart of the study. The demographic characteristics of the participants and the medication used are presented in Table 1. Shortness of breath and inability to maintain pace were the common reasons for ISWT termination. In contrast, perception of exertion ≥ 15 (on the RPE scale) and reaching 85% predicted HR reserve was the common reason for ISWT-T termination (see Table 1).

**Table 1:** Demographic characteristics of the participants.

Characteristic	Value
Age (years)	66.3 ± 7.3
Height (m)	1.73 ± 0.1
Weight (kg)	86.6 ± 10.1
BMI (kg/m^2^	28.8 ± 2.6
WC (cm)	102.6 ± 9.6
LL (cm)	93.1 ± 5.3
HR rest (bpm) before doing ISWT	69 ± 15
HR rest (bpm) before doing ISWT-T	67 ± 14
SBP resting (mmHg) before doing ISWT	129 ± 15
SBP resting (mmHg) before doing ISWT-T	132 ± 11
DBP resting (mmHg) before doing ISWT	78 ± 8
DBP resting (mmHg) before doing ISWT-T	79 ± 5
Diagnosis	
Valve replacement (%)	7.7
CABG (%)	53.8
Dilated cardiomyopathy (%)	7.7
Angina (%)	23.1
PTCA with stent (%)	15.4
Atrial fibrillation (%)	15.4
Reasons for test termination for ISWT	
SOB (%)	38.5
RPE ≥ 15 (%)	15.4
Achieved 85% predicted HR reserve (%)	30.8
Unable to maintain pace (%)	38.5
Severe leg pain (%)	7.7
Reasons for test termination for ISWT-T	
SOB (%)	23.2
RPE ≥ 15 (%)	30.8
Achieved 85% predicted HR reserve (%)	30.8
Unable to maintain pace (%)	15.4
Sever leg pain (%)	7.7
Medication	
β blockers (%)	38.5
Ca Channel blockers (%)	7.7
ACE inhibitors (%)	61.5
Nitrates (%)	38.5
Antiplatelet (%)	69.2
Anticoagulant (%)	23.1
Statin (%)	53.8
Digoxin (%)	7.7

Values are presented as mean and standard deviation or percentages when appropriate. *WC* waist circumference, *LL* leg length, *HR* heart rate, *SBP* systolic blood pressure, *DBP* diastolic blood pressure, *SOB* shortness of breath, *RPE* rate of perceived exertion, *CABG* coronary artery bypass grafting, *PTCA* percutaneous transluminal coronary angioplasty, *ACE* angiotensin-converting enzyme.

### Comparison in cardiopulmonary response between ISWT and ISWT-T

Paired sample *t-*test was used to compare the physiological responses between ISWT and ISWT-T. There were no significant differences in the cardiopulmonary responses between ISWT and ISWT-T except for VO_2PEAK_ [*t*_12_ = − 2.22, *p* = 0.05] (Table 2).

**Table 2:** Comparison of the cardiopulmonary responses between ISWT and ISWT-T.

Variable	ISWT (mean ± SD)	ISWT-T (mean ± SD)	*p* value
HR peak (bpm)	125 ± 16	125 ± 16	0.5
SBP post-test (mmHg)	172 ± 23	169 ± 21	0.2
DBP post-test (mmHg)	87 ± 11	85 ± 9	0.2
Distance (m)	559.2 ± 187.9	544.8 ± 195.1	0.3
RF	35 ± 8	35 ± 9	0.5
VT peak (liter)	1.9 ± 0.5	1.8 ± 0.5	0.1
VE peak (L/min)	63.1 ± 18.4	58.7 ± 16.1	0.1
RER	0.97 ± 0.1	0.94 ± 0.2	0.3
VO_2PEAK_ (ml/kg/min)	25.4 ± 5.8	23.7 ± 5.1	0.05

*ISWT* incremental shuttle walking test, *ISWT-T* incremental shuttle walking test on a treadmill, *HR* heart rate, *SBP* systolic blood pressure, *DBP* diastolic blood pressure, *RF* respiratory frequency, *VT* tidal volume, *VE* minute ventilation, *RER* respiratory exchange ratio, *VO*_*2peak*_ peak volume of oxygen.

### Factors related to the achieved distance in ISWT and ISWT-T

**Figure 2:**
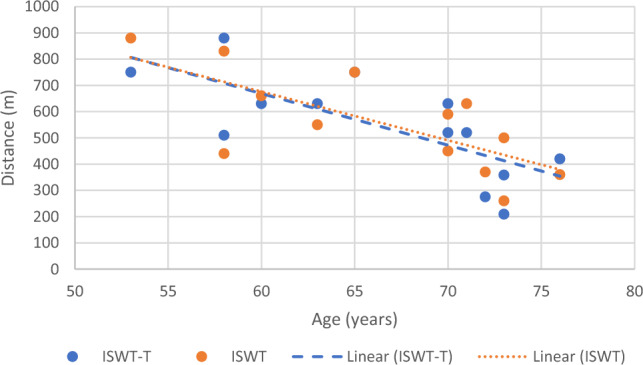
Correlation between age and achieved distance.

**Figure 3:**
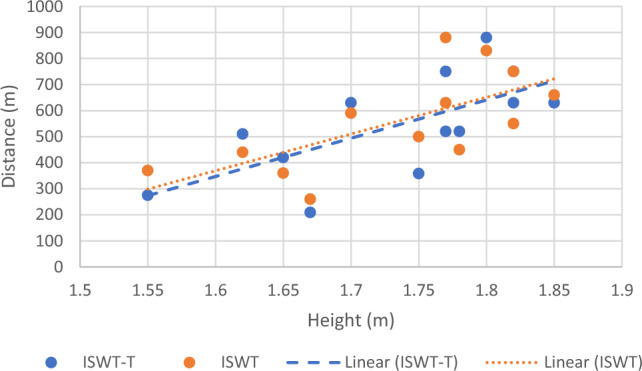
Correlation between height and achieved distance.

**Figure 4:**
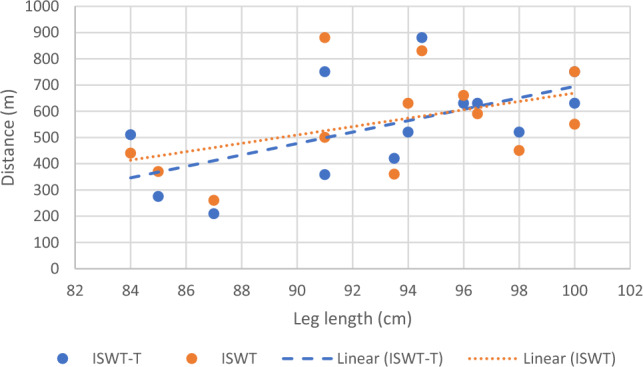
Correlation between leg length and achieved distance.

Correlational analysis was used to assess the factors associated with the achieved distance during each test. Age was strongly and inversely correlated with distance achieved during ISWT and ISWT-T (Fig. 2), and height was moderately correlated with the distance achieved during ISWT and ISWT-T (Fig. 3) (Table 3). Leg length was only correlated moderately with distance achieved during ISWT-T (Fig. 4).

**Table 3:** Factors associated with distance achieved during each test.

Variable	Distance achieved ISWT (*r*)	Distance achieved ISWT-T (*r*)
Age	− 0.72*	− 0.73*
Height	0.68*	0.68*
Weight	0.46	0.41
BMI	− 0.16	− 0.23
WC	0.5	0.11
LL	0.45	0.59*

*p-value ≤ 0.05.

## Discussion

The current study investigated and compared the cardiopulmonary responses between ISWT and ISWT-T as a primary aim and the factors related to the achieved distance of each test as a secondary aim. The results showed that both tests produced, to some extent, similar cardiopulmonary responses except for the VO_2PEAK_. Additionally, age and height were strongly associated with the distance achieved during both tests, with leg length as a factor moderately associated with distance achieved on ISWT-T only. These results indicate that ISWT produces higher cardiorespiratory and metabolic demands than performing on a treadmill (ISWT-T) in patients' phase IV cardiac rehabilitation. Therefore, based on the results of the current study, the cardiopulmonary metabolic demands between the two tests are different, and people with longer leg lengths may achieve better distance on ISWT-T. This needs to be confirmed in a more extensive study.

Laboratory-based cardiopulmonary exercise testing is known as the gold standard for assessing exercise tolerance. However, conducting this test can be time-consuming in clinical practice and may require expensive equipment to be appropriately conducted regularly. Thus, graded externally paced exercise tolerance tests such as ISWT provide an easy, reliable, safe^13^ and valid assessment tool for people enrolled in cardiac rehabilitation programs^14–16^. ISWT was also strongly associated with cardiopulmonary exercise testing^14^, which allows the measurement of physical fitness based on the distance achieved during the assessment^17^. This assessment at the beginning exercise training program helps to tailor the appropriate exercise intensity and serves as an outcome measure to assess the effectiveness of the exercise training program^18^.

There was no difference in the cardiovascular response between the two tests in the current study, including peak HR, systolic BP, and diastolic BP (Table 2). In parallel to the current study's findings, a previous study on pulmonary rehabilitation patients (n = 11) showed no difference at the end of test HR between performing ISWT and ISWT-T^4^. Similarly, no difference in peak HR was reported when ISWT and ISWT-T were performed on (n = 55) healthy adults^3^. In contrast, elevated peak HR, systolic BP, and diastolic BP were reported after the treadmill exercise test compared to ISWT in (n = 55) people with intermittent claudication^19^. However, it must be noted that in the latter study, the exercise testing protocol performed on the treadmill (walking speed 3.2 km h^−1^ at 12% gradient) was different from the ISWT protocol, which may explain the elevation in cardiovascular response after the treadmill testing.

Several studies investigated the difference in walking distances between field tests and performing similar tests on a motorized treadmill. Six-minute walking test (6MWT), as a similar but self-paced test, was compared with 6MWT performed on a treadmill in (n = 19) people with chronic obstructive pulmonary disease (COPD). The results showed a substantial decrease in achieved distance when performed on a treadmill (509 ± 66 m vs 407 ± 86 m, respectively)^5^. Similarly, the same findings were reported in another study among recipients of pulmonary rehabilitation programs, indicating a reduction in 6MWT achieved distance when performed on a treadmill^20^.

A controversial finding was reported in a study among people with COPD indicating longer distance achieved in the shuttle walking test on a treadmill (435 ± 167 m) than on the corridor (354 ± 156 m)^21^. Additionally, when ISWT and ISWT-T were performed on (n = 55) healthy people, the achieved distance was longer in ISWT-T^3^. In the current study, no significant difference in achieved distances between ISWT and ISWT-T (Table 2). However, it must be noted that the mean achieved distance on ISWT was longer but not statically significant than ISWT-T. The variation and controversy in findings between studies can be explained due to differences in methodology, the functional capacity assessment used and different groups of people. One possible explanation for the shorter distance when tests are performed on a treadmill is the lack of familiarity with walking, especially in older people^22^. ISWT mimic walking in routine daily life; thus, people are more familiar with walking than on a treadmill. Also, speeding up in ISWT requires minimal conscious effort to increase the speed, but in ISWT-T, speeding up requires more conscious and additional voluntary movement^20^.

Regarding the total distance walked during ISWT, reference values were created from patients with cardiovascular diseases entering exercise-based cardiac rehabilitation. Given the mean age of the participants (66.3 ± 7.3 years) and that 87% of them were male, the current study’s total distance walked during ISWT (559.2 ± 187.9) is close to the 90th percentile of the reference values reported by Cardoso et al.^23^. This indicates that the achieved distance is commonly reported by people in the same age category.

In the current study, the pulmonary response in both tests was similar (Table 2) except for VO_2PEAK_. First, it is worth noting that there might be a possible explanation for the increased metabolic demands during ISWT. The deceleration acceleration stage may impose additional energy demands from the musculoskeletal system to the cardiopulmonary system^24^. The deceleration stage occurs when reaching the cone at the end of the shuttle to allow turning around the cone, whereas the acceleration stage starts after turning around the cone at the beginning of a shuttle. Thus, the changes in direction during ISWT may explain the marginal difference in energy requirement compared to straight-line walking during ISWT-T. However, this marginal difference may not be clinically relevant, as most of the physiological responses were similar between the two tests. It is also noteworthy, that it has been suggested that turning during ISWT has no influence on the performance of patients with stable cardiovascular diseases and the influence may be limited to those with reduced mobility and orthopaedic disorders^25^.

Regarding the clinical relevance of the difference, ISWT and ISWT-T are closely related as both provoked similar cardiovascular responses. Additionally, ISWT-T may provide better advantages when physical space is limited and the less need for transportation of monitoring equipment when needed and close supervision of the participants^20^. Although there was a statistically significant difference in VO_2PEAK_ between the two tests, clinically, this difference is not important given the similarities between the two tests in other cardiopulmonary and physiological parameters. Therefore, ISWT-T may provide an alternative when there is a limitation in physical space. In the same context, ISWT also provides an alternative when laboratory access is limited and testing in an open wide area is more favoured to minimize severely infectious viral transmission, similar to what occurred during the COVID-19 pandemic.

Appropriate risk stratification and functional capacity assessment using graded exercise testing are fundamental to exercise training programs and make them safer for cardiac patients. Thus, exercise testing should be standardized and performed without estimation based on previously reported values from other populations. Previous studies had shown controversial findings when the metabolic demands between ISWT and ISWT-T were compared. For example, higher energy cost per meter was reported in cardiac rehabilitation patients in ISWT-T (3.22 ± 0.55 J/kg/m) when compared to ISWT (3.00 ± 0.41 J/kg/m) at the early stages of the tests^7^. However, this was reversed at higher speeds as ISWT had a greater energy cost per meter than ISWT-T. Thus, the energy cost of walking on a treadmill (ISWT-T) did not reflect ISWT. This is similar to the current study's findings. Estimation of the metabolic energy cost for ISWT published by the American College of Sports Medicine (ACSM) should be avoided when the ISWT is performed on a treadmill and for cardiac disease patients^2^. This emphasizes that estimation of metabolic demands should be avoided, and direct measurement with the available tools should be utilized either for ISWT or ISWT-T. Additionally, once either measurement is used, consistency in re-assessment should be performed, and training intensities should be set according to the loads obtained and not estimated from either ISWT or ISWT-T.

To the best of knowledge, this is the first study which investigated the factors associated with the distance achieved in ISWT and ISWT-T in people with cardiovascular diseases. In a study among a community-based cardiac rehabilitation center, height explained 55% of the variance in ISWT performance^26^. A previous study reported a predictive model which included age, height and weight were moderately correlated with the distance achieved in ISWT in (n = 131) older healthy Brazilian adults^27^. Similarly, in the current study, age and height were strongly correlated with achieved distance in both ISWT and ISWT-T (Figs. 2, 3) (see Table 3). This indicates that older people may have shorter distances on both tests, whereas taller people may achieve longer distances. The inverse association between age and achieved distance can be explained due to gradual reduction in muscle mass, muscle strength, and maximal oxygen uptake^28^.

Surprisingly Leg length was only significantly correlated with achieved distance on ISWT-T (Fig. 4). Knowing that stride length is a major predictor of gait speed, thus longer distances are achieved^29^. A possible explanation for the association between height and achieved distance can be attributed to longer stride length among taller people^27^. Previous studies reported similar findings to the current study. For example, height was also strongly associated with distance achieved in 6MWT^30,31^. In a similar context, one of the former studies found no significant association between 6MWT (performed in the hallway) and leg length, parallel to the current study's finding in ISWT^30^. There is a possible explanation for the association between Leg length and ISWT-T, which can be due to the need to keep up with the treadmill's speed via taking faster strides, which eventually increases walking speed. This is probably more manifested in ISWT-T because of the extra voluntary movement needed to increase the treadmill's speed.

There are several limitations in the current study. It is worth noting that the current sample included 13 people with cardiovascular diseases; thus, there might be a possibility of finding a clinically significant difference between the two tests if more patients were recruited. Most of the participants in this study were male, limiting the generalizability of this study. Thus, further research is warranted in a larger sample with equal recruitment of males and females in cardiac disease patients to investigate further the cardiopulmonary response and metabolic demands differences between ISWT and ISWT-T.

In conclusion, the cardiopulmonary response between ISWT and ISWT-T were similar; but the metabolic demands indicated by peak volume of oxygen were higher when performing ISWT in the hallway. However, the difference in peak volume of oxygen is marginal and not clinically relevant. Therefore, performing the test on the treadmill may provide an alternative when physical space is limited and vice versa when robust physical distancing precautions are in place. Age and height are strongly associated with performing the test with and without a treadmill, whereas leg length was moderately associated with performing the test on a treadmill only. To avoid underestimation or overestimation of functional capacity, exercise training should be based on actual assessment and measurement during either ISWT or ISWT-T. The latter could be used as an alternative in physical space limitation without estimation from predetermined values.

### Supplementary Information

Supplementary Information 1.

Supplementary Information 2.

## Data Availability

All data generated or analyzed during this study are included in this published article [and its supplementary information files].

## Abbreviations

ACE: Angiotensin converting enzyme
ACSM: American College of Sports Medicine
BMI: Body mass index
CABG: Coronary artery bypass grafting
CVD: Cardiovascular diseases
DBP: Diastolic blood pressure
HR: Heart rate
ISWT: Incremental shuttle walking test
ISWT-T: Incremental shuttle walking test on a treadmill
LL: Leg length
6MWT: 6-Minutes walking test
PTCA: Percutaneous transluminal coronary angioplasty
RPE: Rate of perceived exertion
SBP: Systolic blood pressure
SPSS: Statistical package for social sciences
SOB: Shortness of breath
VE: Minute ventilation
VO2_PEAK_: Peak volume of oxygen
VO2: Volume of oxygen
VT: Tidal volume
WC: Waist circumference
